# Association of serum copeptin and urinary uromodulin with kidney function, blood pressure and albuminuria at 6 weeks post-partum in pre-eclampsia

**DOI:** 10.3389/fcvm.2024.1310300

**Published:** 2024-03-04

**Authors:** David A. Jaques, Anne Dufey Teso, Grégoire Wuerzner, Begona Martinez De Tejada, Marika Santagata, Véronique Othenin Girard, Bénédicte Le Tinier, Antoinette Pechere Bertschi, Belen Ponte

**Affiliations:** ^1^ Service of Nephrology and Hypertension, Geneva University Hospitals, Geneva, Switzerland; ^2^ Service of Nephrology and Hypertension, Lausanne University Hospital and University of Lausanne, Lausanne, Switzerland; ^3^ Division of Obstetrics, Department of Pediatrics, Gynecology and Obstetrics, Geneva University Hospitals, Geneva, Switzerland; ^4^ Faculty of Medicine, University of Geneva, Geneva, Switzerland

**Keywords:** preeclampsia, post-partum, organ damage, hypertension, albuminuria, proteinuria, uromodulin, copeptin

## Abstract

**Background:**

Preeclampsia (PE) is associated with subsequent higher risk of cardiovascular and kidney disease. Serum copeptin, as a proxy for vasopressin, and urinary uromodulin, were associated with PE physiopathology and kidney functional mass respectively. We describe concentrations of these proteins in the post-partum period and characterize their association with persistent hypertension (HTN) or albuminuria.

**Methods:**

Patients with PE and healthy controls with uncomplicated pregnancy were prospectively included at two teaching hospitals in Switzerland. Clinical parameters along with serum copeptin and urinary uromodulin were measured at 6 weeks post-partum. PE patients were further characterized based on presence of HTN (defined as either systolic BP (SBP) ≥140 mmHg or diastolic (BP) ≥90 mmHg) or albuminuria [defined as urinary albumin to creatinine ratio (ACR) ≥3 mg/mmol].

**Results:**

We included 226 patients with 35 controls, 120 (62.8%) PE with persistent HTN/albuminuria and 71 (37.1%) PE without persistent HTN/albuminuria. Median serum copeptin concentration was 4.27 (2.9–6.2) pmol/L without differences between study groups (*p* > 0.05). Higher copeptin levels were associated with higher SBP in controls (*p* = 0.039), but not in PE (*p* > 0.05). Median urinary uromodulin concentration was 17.5 (7.8–28.7) mg/g with lower levels in PE patients as compared to healthy controls (*p* < 0.001), but comparable levels between PE patients with or without HTN/albuminuria (*p* > 0.05). Higher uromodulin levels were associated with lower albuminuria in PE as well as control patients (*p* = 0.040).

**Conclusion:**

Serum copeptin levels at 6 weeks post-partum are similar between PE patients and healthy controls and cannot distinguish between PE with or without residual kidney damage. This would argue against a significant pathophysiological role of the vasopressin pathway in mediating organ damage in the post-partum period. On the opposite, post-partum urinary uromodulin levels are markedly lower in PE patients as compared to healthy controls, potentially reflecting an increased susceptibility to vascular and kidney damage that could associate with adverse long-term cardiovascular and kidney outcomes.

## 1 Introduction

Preeclampsia (PE) is defined as new onset hypertension (HTN) occurring after 20 weeks of gestation associated with damage to at least one organ (1). Overall, PE is thought to affect 5%–8% of all pregnancies. In most cases, resolution of organ damage is expected in the few weeks following delivery (2). However, some women have persistent HTN and albuminuria, as well as a subsequent higher risk of cardiovascular and kidney disease (3–5).

Vasopressin plays a central role in osmotic and blood pressure (BP) homeostasis. As vasopressin is subject to degradation by trophoblastic enzymes during pregnancy, serum copeptin is used as a surrogate for its secretion in this setting (6). While copeptin has been shown to increase throughout normal pregnancy, comparatively higher levels have been observed in patients who subsequently developed PE (7). Copeptin could thus improve PE prediction as early as 6 weeks into pregnancy (8). Moreover, several pathways linking vasopressin to PE pathophysiology have been proposed and chronic infusion of vasopressin could induce a PE phenotype in pregnant mice (8). Finally, aside from pregnancy, elevated copeptin levels have been associated with adverse cardiovascular and kidney outcomes in clinical as well as epidemiological studies (9–13).

Uromodulin, the most abundant protein in normal urine, is exclusively synthetized by epithelial cells of the thick ascending loop of Henle in renal tubules (14). Physiological functions of uromodulin include protection against kidney stones and urinary tract infections as well as regulation of tubular sodium handling and blood pressure (15–19). Beyond its biological functions, urinary uromodulin also serves as a biomarker of functional tubular mass and low levels have been associated with alteration as well as faster decline of kidney function (19–21).

Overall, the course and clinical implications of copeptin and uromodulin levels after PE are largely unknown. This question is of importance as it could shed light onto pathophysiological mechanisms of organ damage after PE as well as unveil potential therapeutic targets to mitigate the risk of developing long term kidney and cardiovascular diseases. As such, in this study, we wished to (i) compare concentrations of serum copeptin and urinary uromodulin at 6 weeks post-partum between PE and normal pregnancy as well as (ii) describe the association between these proteins and kidney function, BP as well as albuminuria.

## 2 Materials and methods

### 2.1 Participants, study design and procedures

We performed a retrospective cross-sectional analysis of a prospective cohort study including women with PE as well as healthy controls. The original study was conducted in two tertiary teaching hospitals: “Hôpitaux Universitaires de Genève” (HUG), Geneva, Switzerland and “Centre Hospitalier Universitaire Vaudois” (CHUV), Lausanne, Switzerland.

The present study thus included a PE and a control group. All participants were screened after delivery. Regarding the PE group, inclusion required a diagnosis of PE during pregnancy. Exclusion criteria were (i) unwilling to participate, (ii) use of anti-inflammatory drugs and (iii) any known cardiac, endocrine, or renal disease. The control group consisted of healthy women who had an uncomplicated normotensive pregnancy with delivery at term. Exclusion criteria were (i) history of PE during a prior pregnancy, (ii) history of HTN and (iii) proteinuria on dipstick.

If enrolled, participants were evaluated at 6 weeks post-partum with clinical as well as biological parameters. The timing of this visit was chosen to coincide with the gynecologic follow-up routinely performed after delivery and reimbursed by the insurances. Medical history and physical examination were recorded. Office BP was measured according to the European Society of Hypertension guidelines (22). After 5 min of rest, 2 BP measurements were done at 1 min interval on the same arm with a proper cuff size and a validated automated device (Omron HEM-907-E; British Hypertension Society Grade A/A). Arithmetic mean of the 2 values were calculated. Creatinine was measured using the IDMS-traceable Jaffe kinetic compensated method. Estimated glomerular filtration rate (eGFR) was calculated using the 2009 Chronic Kidney Disease-Epidemiology Collaboration (CKD-EPI) formula (23). The urinary albumin to creatinine ratio (ACR) was calculated as albumin/creatinine and expressed as mg/mmol. Serum copeptin (CT-proAVP pmol/L) was measured in batch at the single time point of 6 weeks post-partum on frozen −80° plasma-EDTA samples using a sandwich immunoluminometric assay in Aarau Hospital, Aarau, Switzerland, as previously described (Thermo Fischer Scientific, Brahms GmbH, Hennigsdorf, Germany) (24). Serum copeptin is expressed as pmol/L. Urinary uromodulin concentration was measured by ELISA as described previously (25). Urinary uromodulin was normalized to creatininuria and expressed as mg/g. Human uromodulin (stock solution 100 mg/ml; Millipore) was used for the standard curve. The uromodulin ELISA has a sensitivity of 2.8 ng/ml, a linearity of 1.0, an interassay variability of 3.3%, and an intra-assay variability of 5.5%.

### 2.2 Definitions

PE was defined according to the International Society for the Study of Hypertension in Pregnancy 2014, as new-onset hypertension after 20 weeks of gestation associated with at least one end-organ damage (1). Pre-gestational HTN as well as pre-gestational diabetes mellitus (DM) were defined based on associated medications or known medical history. At 6 weeks post-partum, HTN was defined as either SBP ≥ 140 mmHg or DBP ≥ 90 mmHg and albuminuria was defined as ACR ≥ 3 mg/mmol.

### 2.3 Statistical analysis

Continuous variables were expressed as mean ± standard deviation (SD) or median (interquartile range) according to distribution while categorical variables were expressed as number and relative frequencies. Normality of distribution was assessed graphically. Patient's characteristics were compared between groups using Student's *T* test or ANOVA for continuous variables as well as Chi2 for categorical variables. Linear regression models were used to describe associations with continuous dependent variables. Continuous variables were log-transformed when appropriate. Potential interactions between independent variables were tested using contrasts of marginal linear predictions. Sensitivity analyses were conducted with multivariate adjustment to account for potential confounders [PE status, age, ethnicity and body mass index (BMI)]. Data were considered missing completely at random and patients with any missing value were thus excluded from regression models. A two-sided *p*-value <0.05 was considered significant in descriptive analyses as well as regression models. Statistical analyses were conducted using STATA version 17 (StataCorp, 4905 Lakeway Drive, College Station, Texas 77845 USA).

### 2.4 Ethics

This study was approved by the local ethics committee “Commission cantonale d’éthique de la recherche” (CCER), Geneva, Switzerland and was performed according to the Declaration of Helsinki. Written informed consent was obtained from each participant.

## 3 Results

**Figure 1:**
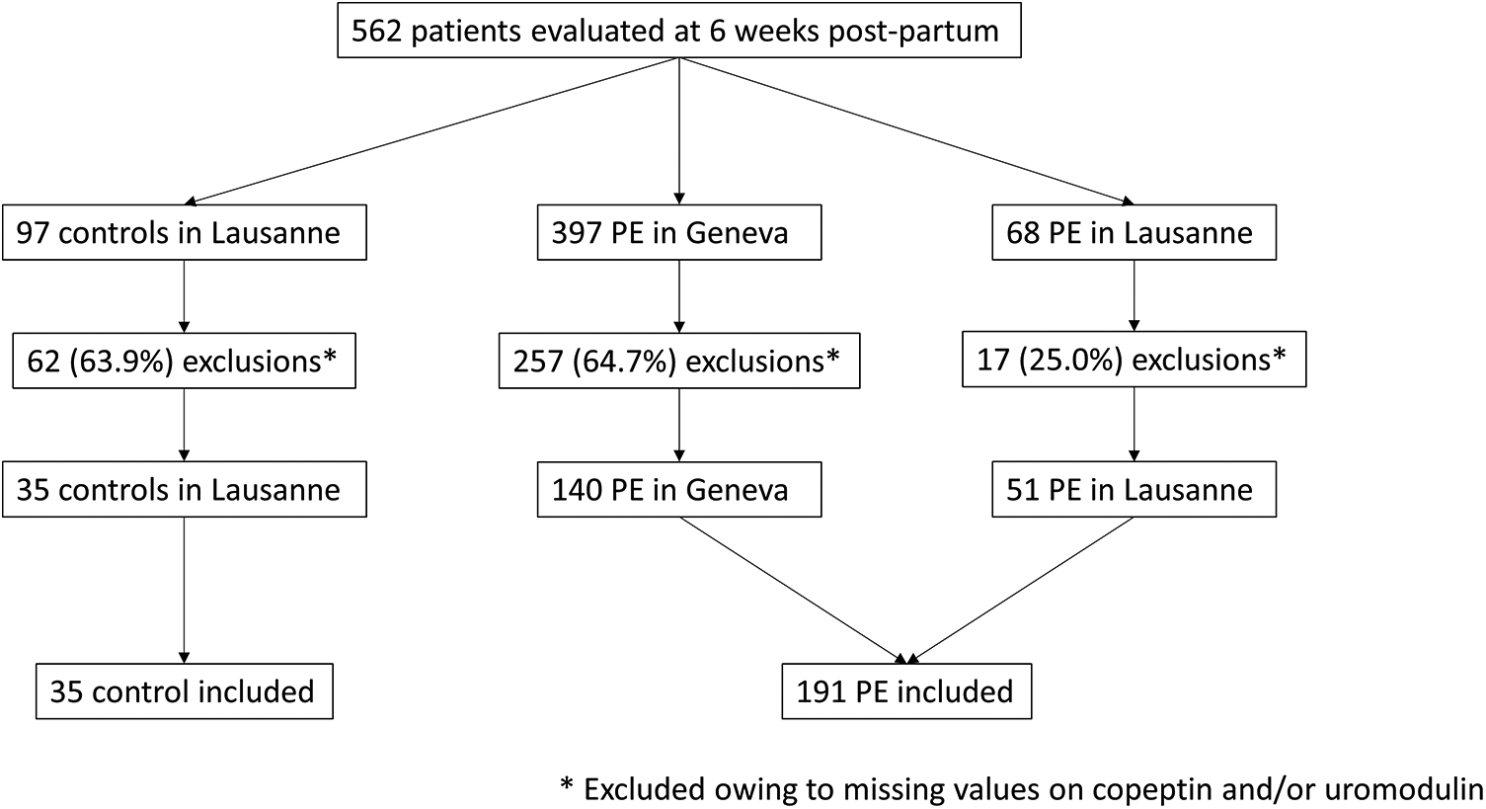
Study flowchart. PE, preeclampsia.

In total, 562 patients were evaluated at 6 weeks post-partum with 97 healthy controls and 465 PE patients (Figure 1). Among those, 62 control and 274 PE were excluded owing to missing data on serum copeptin and/or urinary uromodulin. Baseline characteristics of included and excluded patients according to PE status are given in Supplementary Table S1. Regarding healthy controls, characteristics of included and excluded women were similar between groups. Regarding PE patients, excluded patients had higher BMI and higher prevalence of gestational DM and prior PE. Other characteristics were similar between groups.

**Figure 2:**
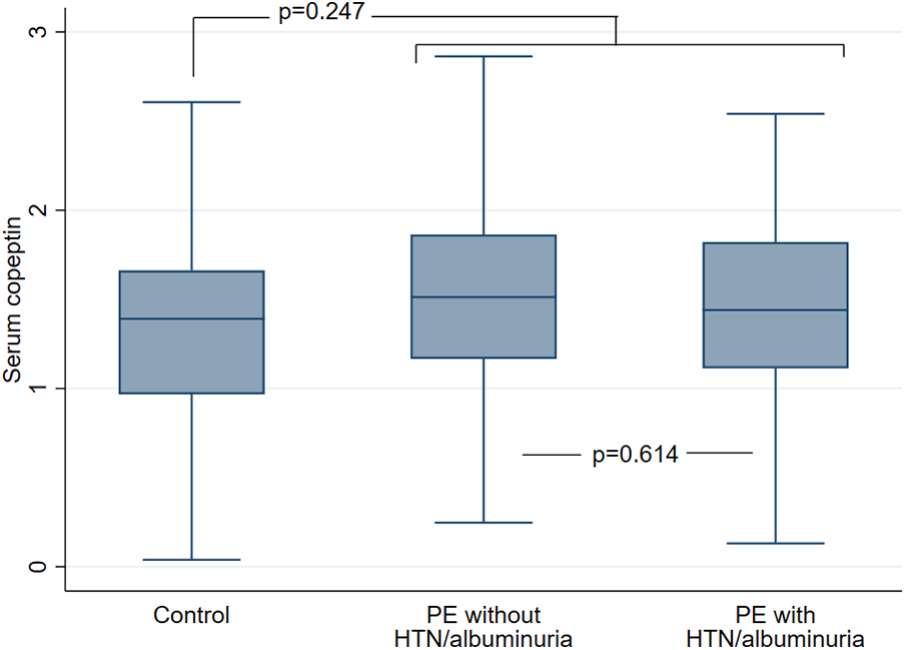
Serum copeptin concentration (pmol/L, log-transformed) at 6 weeks post-partum according to PE status and presence or absence of persistent HTN or albuminuria. Data are presented as median, 25th and 75th percentiles as well as upper and lower adjacent values. Comparison between control participants (*N* = 35) and PE patients as a whole (*N* = 191). *T*-test *p* value 0.247. Comparison between PE patients with (*N* = 120) and without (*N* = 71) HTN/albuminuria. *T*-test *p* value 0.614. PE, preeclampsia; HTN, hypertension.

**Figure 3:**
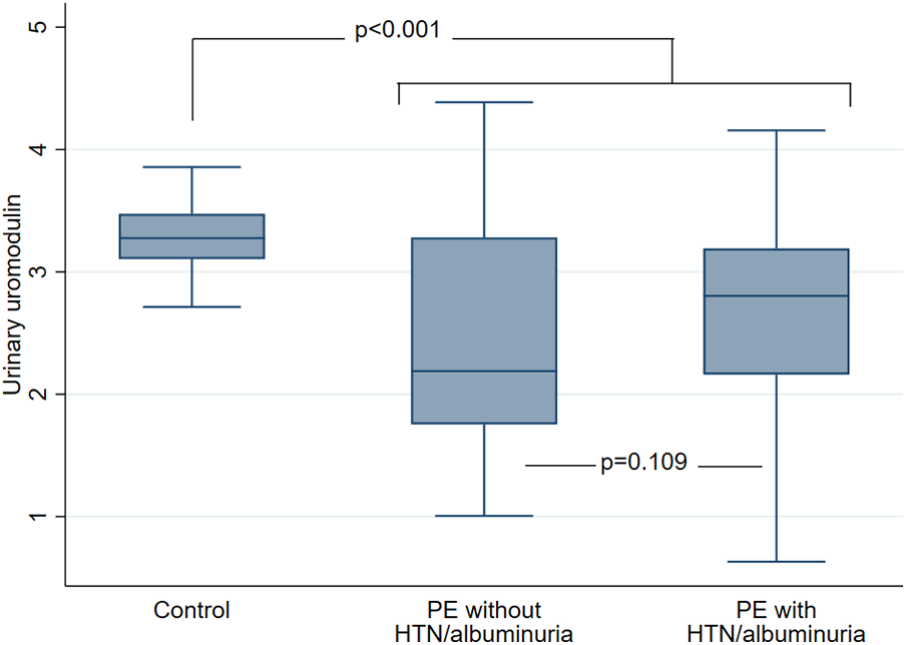
Urinary uromodulin concentration (mg/g, log-transformed) at 6 weeks post-partum according to PE status and presence or absence of persistent HTN or albuminuria. Data are presented as median, 25th and 75th percentiles as well as upper and lower adjacent values. Comparison between control participants (*N* = 35) and PE patients as a whole (*N* = 191). *T*-test *p* value <0.001. Comparison between PE patients with (*N* = 120) and without (*N* = 71) HTN/albuminuria. *T*-test *p* value 0.109. PE, preeclampsia; HTN, hypertension.

Eventually, 226 patients (191 PE and 35 controls) were included in the present study. Mean age was 32.6 ± 5.4 with 153 (67.7%) Caucasians. Among 191 patients with PE, 120 (62.8%) had persistent HTN or albuminuria at 6 weeks post-partum while 71 (37.1%) did not. Baseline characteristics according to PE status and the presence or absence of HTN/albuminuria are given in Table 1. As compared to healthy controls, patients with PE were less frequently Caucasians, had higher BMI, were more frequently smokers, had shorter gestational time and higher eGFR. As compared to PE patients without HTN/albuminuria as well as healthy controls, PE patients with HTN/albuminuria had higher SBP, DBP and ACR values. Median serum copeptin concentration was 4.27 (2.9–6.2) pmol/L without differences between study groups (Table 1; Figure 2). Median urinary uromodulin concentration was 17.5 (7.8–28.7) mg/g with lower levels in PE patients as compared to healthy controls, but comparable levels between PE patients with or without HTN/albuminuria (Table 1; Figure 3). After adjustment for age, ethnicity and BMI, urinary uromodulin concentration remained significantly lower in PE patients as compared to healthy controls (*p* = 0.001).

**Table 1:** Baseline characteristics at 6 weeks post-partum according to PE status and the presence or absence of persistent HTN or albuminuria.

	Control (*N* = 35)	PE without HTN/albuminuria (*N* = 71)	PE with HTN/albuminuria (*N* = 120)	*P* value
	Clinical characteristics			
Age (years)	32.7 ± 5.1	31.4 ± 5.7	33.2 ± 5.2	0.079
Ethnicity				
Caucasian	35 (100%)	48 (67.6%)	70 (58.3%)	** **
Afro-American	0 (0%)	11 (15.4%)	27 (22.5%)	**0**.**001**
Hispanic	0 (0%)	11 (15.4%)	20 (16.6%)	** **
Asian	0 (0%)	1 (1.4%)	3 (2.5%)	** **
BMI (kg/m^2^)	24.4 ± 4.3	29.1 ± 4.7	28.9 ± 6.5	**<0**.**001**
Smoker	1 (3.3%)	21 (29.5%)	11 (9.4%)	**<0**.**001**
Essential HTN	0 (0%)	3 (4.2%)	11 (9.3%)	0.094
Gestational DM	0 (0%)	7 (9.8%)	7 (6.0%)	0.143
Primiparity	18 (60.0%)	46 (65.7%)	74 (63.7%)	0.861
Gestational weeks	40 (38–40)	38 (36–40)	36 (33–38)	**<0**.**001**
Prior PE	0 (0%)	4 (5.6%)	10 (8.6%)	0.217
	Evaluation at 6 weeks post-partum			
SBP (mmHg)	112.0 ± 11.6	115.4 ± 9.3	127.3 ± 15.4	**<0**.**001**
DBP (mmHg)	71.2 ± 10.3	76.3 ± 6.5	88.3 ± 11.9	**<0**.**001**
HTN^a^	1 (2.8%)	0 (0%)	88 (73.3%)	**<0**.**001**
eGFR (ml/min/1.73 m^2^)	97.3 ± 16.2	111.9 ± 18.6	108.4 ± 18.8	**<0**.**001**
ACR (mg/mmol)	1.0 (0.6–2.1)	1.2 (0.7–1.6)	3.7 (1.6–7.6)	**<0**.**001**
Albuminuria^b^	7 (20.0%)	0 (0%)	72 (60.0%)	**<0**.**001**
	Serum copeptin and urinary uromodulin at 6 weeks post-partum			
Copeptin (pmol/L)	4.0 (2.6–5.2)	4.5 (3.2–6.4)	4.2 (3.0–6.1)	0.451
Uromodulin (mg/g)	26.4 (22.3–32.2)	8.9 (5.7–26.6)	16.5 (8.6–24.3)	**<0**.**001**

PE, preeclampsia; HTN, hypertension; BMI, body mass index; DM, diabetes mellitus; SBP, systolic blood pressure; DBP, diastolic blood pressure; eGFR, estimated glomerular filtration rate; ACR, urinary albumin to creatinine ratio.

*P* values indicated in the table apply to comparisons between the three presented groups.

Bold values indicate *p* < 0.05.

^a^ Defined as SBP ≥ 140 mmHg or DBP ≥ 90 mmHg.

^b^ Defined as ACR ≥ 3 mg/mmol.

### 3.1 Association of serum copeptin with BP, eGFR and ACR

**Figure 4:**
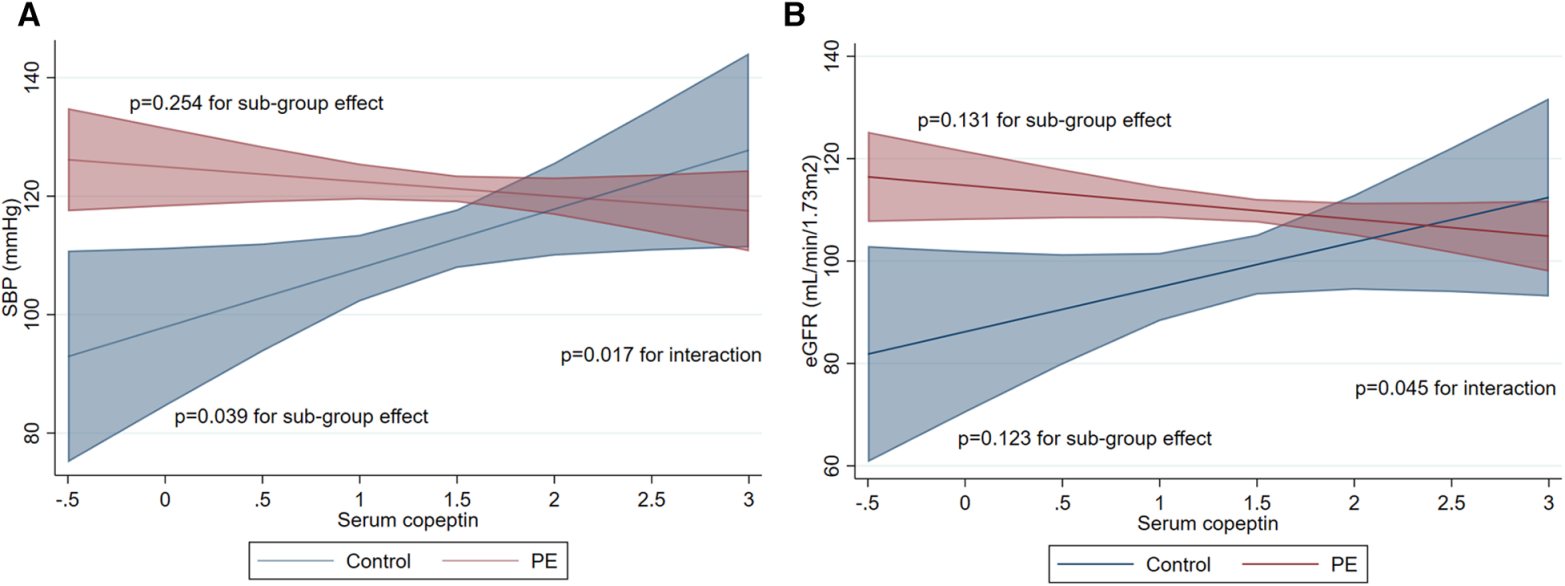
Association of serum copeptin concentration (pmol/L, log-transformed) at 6 weeks post-partum with outcomes according to PE status. (**A**) Association with SBP. Interaction between serum copeptin and PE status is significant with *p* value of 0.017 (contrast of marginal linear predictions) confirming a modification effect. Consequently, sub-group associations for control participants (*N* = 32) and PE patients (*N* = 139) are presented separately with associated *p* values of 0.039 and 0.254 respectively (average marginal effects of multivariate linear regression). (**B**) Association with eGFR. Interaction between serum copeptin and PE status is significant with *p* value of 0.045 (contrast of marginal linear predictions) confirming a modification effect. Consequently, sub-group associations for control participants (*N* = 32) and PE patients (*N* = 186) are presented separately with associated *p* values of 0.123 and 0.131 respectively (average marginal effects of multivariate linear regression). PE, preeclampsia; SBP, systolic blood pressure; eGFR; estimated glomerular filtration rate.

In univariate analysis, copeptin levels were not associated with SBP, DBP, eGFR or ACR (*p* > 0.05 for all). In multivariate analysis accounting for PE status, age, ethnicity, and BMI, copeptin levels were not associated with SBP, DBP, eGFR or ACR (Table 2). However, a significant interaction was present between copeptin levels and PE status in association with SBP and eGFR (*p* = 0.017 and *p* = 0.045 for interaction terms respectively). In control participants, serum copeptin was positively associated with SBP. In PE patients, serum copeptin was not associated with SBP. The differential effect of copeptin levels on SBP according to PE status adjusted for confounders is represented in Figure 4A. In control participants as well as PE patients, serum copeptin was not associated with eGFR. The differential effect of copeptin levels on eGFR according to PE status adjusted for confounders is represented in Figure 4B. No significant interaction was present between copeptin levels and PE status in association with DBP or ACR (*p* > 0.05 for interaction terms).

**Table 2:** Association of serum copeptin and urinary uromodulin with SBP, DBP, eGFR and ACR at 6 weeks post-partum.

	SBP		DBP		eGFR		ACR	
	β (95% CI)	*P* value	β (95% CI)	*P* value	β (95% CI)	*P* value	β (95% CI)	*P* value
Copeptin	−0.5 (−4.5 to 3.4)	0.801	−1.4 (−4.6 to 1.8)	0.388	−1.8 (−5.9 to 2.2)	0.377	0.0 (−0.2 to 0.4)	0.535
Control	9.9 (0.5–19.3)	**0**.**039**			8.7 (−2.3 to 19.8)	0.123		
PE	−2.4 (−6.7 to 1.7)	0.254			−3.3 (−7.6 to 0.9)	0.131		
Uromodulin	1.2 (−1.4 to 4.0)	0.364	0.8 (−1.3 to 3.1)	0.448	3.4 (0.5–6.4)	**0**.**020**	−0.2 (−0.4 to −0.0)	**0**.**040**

SBP, systolic blood pressure; DBP, diastolic blood pressure; eGFR, estimated glomerular filtration rate; ACR, urinary albumin to creatinine ratio; PE, preeclampsia; BMI, body mass index.

Sub-group associations in control and PE patients are presented when interaction with PE status is significant.

All models are adjusted for PE status, age, ethnicity and BMI.

Bold values indicate *p* < 0.05.

### 3.2 Association of urinary uromodulin with BP, eGFR and ACR

**Figure 5:**
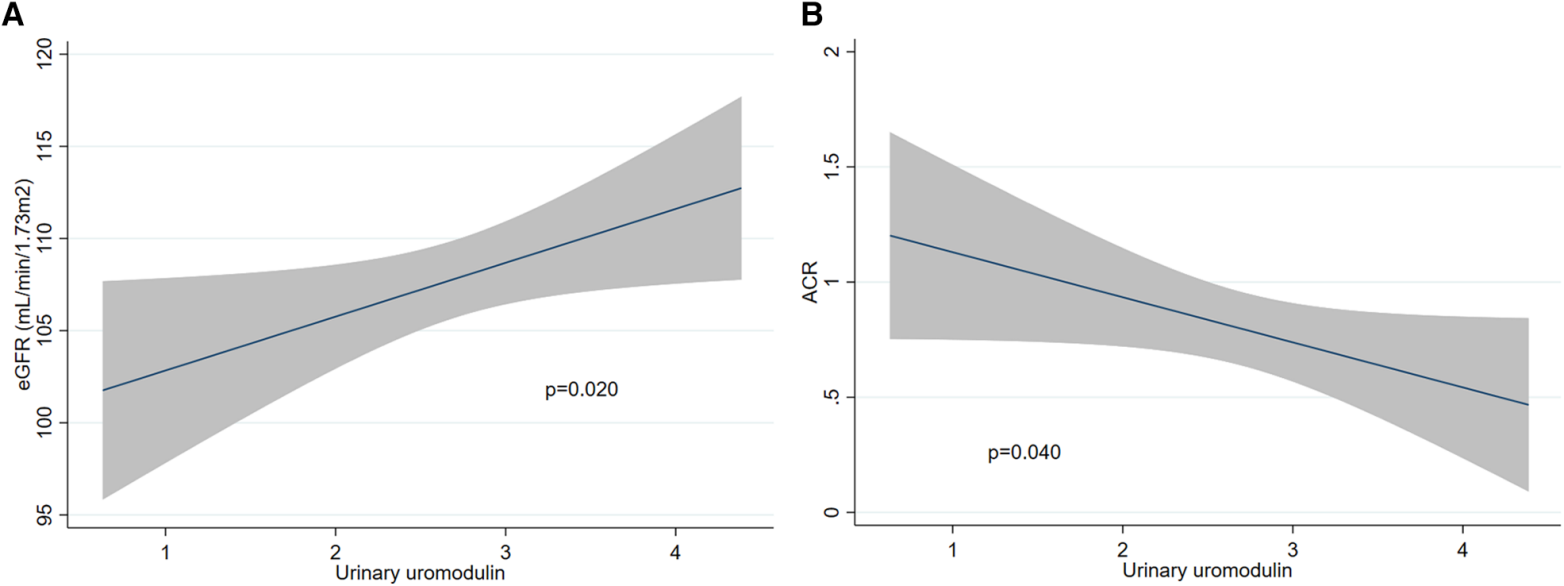
Association of urinary uromodulin concentration (mg/g, log-transformed) at 6 weeks post-partum with outcomes. (**A**) Association with eGFR (ml/min/1.73 m^2^). *N* = 218. *P* value of 0.020 (multivariate linear regression). (**B**) Association with ACR (mg/mmol creatinine, log-transformed). *N* = 218. *P* value of 0.040 (multivariate linear regression). PE, preeclampsia; eGFR, estimated glomerular filtration rate; ACR, urinary albumin to creatinine ratio.

In univariate analysis, uromodulin levels were negatively associated with ACR (β −0.2, 95%CI −0.4 to −0.0, *p* = 0.008) but not with SBP, DBP or eGFR (*p* > 0.05 for all). In multivariate analysis accounting for PE status, age, ethnicity and BMI, uromodulin levels were positively associated with eGFR and negatively with ACR (Table 2). On the opposite, uromodulin levels were not associated with SBP or DBP. The effect of uromodulin levels on eGFR and ACR is represented in Figures 5A,B respectively. No significant interaction was present between uromodulin levels and PE status in association with SBP, DBP, eGFR or ACR (*p* > 0.05 for interaction terms).

## 4 Discussion

In this study, we show that serum copeptin levels at 6 weeks post-partum are similar between PE patients and healthy controls and cannot distinguish between PE patients with or without residual kidney damage. However, at this same time point, urinary uromodulin levels are markedly lower in PE patients as compared to healthy controls.

The use of serum copeptin/vasopressin during pregnancy has gained significant clinical interest in the past years. First, copeptin could serve as an early diagnostic biomarker to detect subsequent PE in pregnant women. In a pivotal study, Santillan et al. performed sequential copeptin measurements during pregnancy course and reported high predictive performances for the development of later PE as early as 6 weeks into pregnancy (8). A recent meta-analysis aggregating available observational studies reporting serum copeptin values among PE and healthy controls found increased copeptin levels prior to PE in all trimesters, irrespective of disease severity and onset (26). Second, beyond a potential diagnostic utility, evidence also indicates a pathogenic role of vasopressin in the development of PE. As such, infusion of vasopressin throughout gestation was demonstrated to induce PE phenotype in mice, including elevated SBP, proteinuria as well as glomerular endotheliosis (8). Subsequent experimental work showed that this was also the case in the absence of placental hypoxia (27). At the same time, it must be remembered that serum copeptin has consistently been associated with adverse clinical outcomes aside from pregnancy. Overall, in clinical observational studies, elevated copeptin was correlated to higher BP, lower eGFR and increased albuminuria (28, 29). Moreover, it was also associated with decline in eGFR over time as well as renal events, cardiovascular events and mortality (9, 13, 29–31). Altogether those data raise the question of a potential implication of vasopressin in mediating organ damage after an episode of PE. Specifically, persistently elevated copeptin levels in the post-partum period could theoretically be implicated in the sub-group of PE patients presenting with persistent renal and cardiovascular damage. Along with offering pathophysiological insights, such a hypothesis would also unveil a potentially attractive therapeutic target to mitigate the risk of long-term organ damage after PE. In the present study, we confirm that PE patients have higher SBP, DBP and ACR than healthy controls at 6 weeks post-partum. Those results are in line with prior evidence reporting an increased risk of HTN as well as subsequent kidney and cardiovascular disease after PE (32–34). We also observed that around two thirds of PE patients had persistent HTN or albuminuria at this time point. Interestingly however, we found that copeptin levels at 6 weeks post-partum were similar between patients with or without prior PE. We observed a median copeptin concentration of 4.27 pmol/L in the entire cohort. This is only slightly higher than what is described in healthy non-pregnant women with median values of 3.2 pmol/L ranging from 1.0 to 14.8 pmol/L (35). This suggests that copeptin levels rapidly decline in the few weeks following delivery in women with PE as well as those with uncomplicated pregnancy. Our results corroborate recent data reporting no difference in urinary copeptin levels at 1–4 years post-partum in patients with and without prior PE (36). Moreover, we show that copeptin levels at 6 weeks post-partum do not discriminate between PE patients with or without persistent organ damage at this time point. Overall, our results would suggest that vasopressin does not play a significant pathophysiological role in mediating organ damage during the post-partum period of PE. Consequently, exogenous manipulation of vasopressin activity via receptors antagonism would likely be vain at this stage of the disease. On a theoretical point of view, it is worth mentioning however that elevated copeptin levels during pregnancy might nevertheless be associated with subsequent post-partum organ damage. This could however not be tested in our cross-sectional study. Finally, while copeptin concentration itself was not associated with BP values, eGFR or ACR overall, we could observe that the occurrence of PE modified some of those associations. Specifically, while copeptin was not associated with SBP in patients with prior PE, we observed a positive association in healthy controls, in line with prior evidence aside from pregnancy (28). A similar modulating effect of PE was observed regarding the relationship between copeptin and eGFR, although clinical interpretation is rendered tedious by the glomerular hyperfiltration described in the post-partum period whether after PE or a healthy pregnancy (37, 38).

During the past decade, experimental as well as clinical evidence provided major insights into the potential roles of uromodulin. Besides physiological considerations, uromodulin can also be seen as a biomarker of tubular mass and function. In epidemiological studies, urinary uromodulin was associated with eGFR as well as kidney volume (21, 39). Moreover, 24 h urinary excretion of uromodulin correlated with known predictors of nephron mass (40). Serum uromodulin was also associated with kidney function in patients with CKD, with greater sensitivity than conventional biomarkers (creatinine, urea, cystatin C) to detect early disease (41, 42). In addition, serum uromodulin could represent an early marker of kidney fibrosis (43). In view of those data, uromodulin could be seen as a surrogate marker of nephron functional mass in heath as well as kidney disease. Interestingly, serum as well as urinary uromodulin also convey prognostic significance as low levels were associated with an increased risk of decline in kidney function and progression to end-stage kidney disease (ESKD) (20, 44). Finally, the role of uromodulin as a biomarker could extend beyond evaluation of kidney function as serum uromodulin could predict cardiovascular events as well as mortality even after adjusting for eGFR as well as cardiovascular risk factors (45). In view of those evidence and given the potential impact of PE on the subsequent renal and cardiovascular prognosis of affected patients, we wished to characterize urinary uromodulin levels in this setting. In the present study, we clearly found that urinary uromodulin levels at 6 weeks post-partum were significantly lower in patients with prior PE as compared to healthy controls. Consequently, a potential pathophysiological hypothesis would be that the lower uromodulin levels in patients suffering from PE reflected a pre-existing increased susceptibility to vascular and kidney damage. Conversely, higher uromodulin levels would reflect a better preserved structural and functional renal reserve (19). Such a protective effect of uromodulin on future events has been shown in type 1 diabetes, where patients with higher serum uromodulin levels were less likely to develop CKD (46). Our findings are also to be related to a prior study, where authors described a transient decrease in urinary uromodulin in hypertensive pregnant women that subsequently normalized in the early post-partum period (47). In view of those results as well as the potential implication of uromodulin in the IL-1β/NLRP3 inflammasome, a contribution of the immunological system in the development of PE could be postulated (48). Alternatively, a reverse causality can be postulated and the occurrence of PE itself could lead to a rapid decrease in uromodulin levels as a reflection of ongoing kidney damage. However, this second hypothesis seems less likely as we could not detect differences in uromodulin levels between PE patients with or without kidney damage. In any case, independently of the pathophysiological mechanism involved, it seems reasonable to hypothesize that the lower post-partum levels of uromodulin after PE could be associated with the adverse long-term cardiovascular and kidney outcomes of this population. Finally, we could also find a direct relationship between uromodulin levels and eGFR as well as ACR, independently of the presence or absence of prior PE. Higher urinary uromodulin concentration was indeed related to lower albuminuria and higher eGFR in our whole cohort, event after adjusting for potential confounders. This last finding is to be related to prior studies in healthy as well as diabetic participants, also describing lower albuminuria and higher eGFR with increasing uromodulin concentration (21, 49, 50).

### 4.1 Limitations

Several limitations apply to our findings. First and most importantly, copeptin and uromodulin were measured at a single time point in the post-partum period and longitudinal variations were not assessed. However, the evolution of copeptin levels during pregnancy is well characterized in prior reports and meaningful variations at later time points during the post-partum period are unlikely given our findings at 6 weeks after delivery. Likewise, BP measurements were also restricted to a single time point and evaluation of BP trajectories over time was not possible. Second, the rather limited number of control participants, while sufficient to test our main hypotheses given the statistically significant results, prevented us to conduct refined sub-group analyses in case of interacting effect. This underlines the difficulty in performing post-partum studies in healthy controls. Moreover, the exclusively Caucasian population of control participants might preclude generalizability of our findings in other settings. Third, BP evaluation relied on office BP measurement as 24 h ambulatory BP monitoring (ABPM) was unavailable in a majority of patients, illustrating difficulties in providing optimal follow-up in the post-partum period. Finally, interpretation of kidney function based on eGFR analysis is rendered difficult given the potentially persisting glomerular hyperfiltration in the post-partum period, especially in patients with prior PE.

## 5 Conclusions

In the present study, we confirm that PE patients have a higher prevalence of HTN and albuminuria than healthy controls at 6 weeks post-partum. Moreover, up to two thirds of PE patients have measurable kidney damage at that point. We also show that serum copeptin levels are similar between patients with or without prior PE and cannot distinguish between PE patients with or without residual kidney damage at this time point. This suggests that copeptin levels rapidly decline in the few weeks following delivery in women with PE as well as those with uncomplicated pregnancy. While not excluding delayed effects, those results would suggest that the vasopressin pathway does not play a significant pathophysiological role in mediating organ damages during the post-partum period following PE. We also found that urinary uromodulin levels at 6 weeks post-partum were significantly lower in patients with prior PE as compared to healthy controls. Consequently, lower uromodulin levels in PE could reflect an increased susceptibility to vascular and kidney damage that could associate with the adverse long-term cardiovascular and kidney outcomes of this population.

## Data Availability

The raw data supporting the conclusions of this article will be made available by the authors, without undue reservation.
