# Supplementary material for: Association of serum copeptin and urinary uromodulin with kidney function, blood pressure and albuminuria at 6 weeks post-partum in pre-eclampsia
Source: Front Cardiovasc Med. 2024 Mar 4;11:1310300. doi: 10.3389/fcvm.2024.1310300 (PMC10945001; doi:10.3389/fcvm.2024.1310300)
Supplement: Supplementary file 1 [file Table1.docx]

Supplementary Material

**Supplementary table 1:** Characteristics of included and excluded patients according to PE status.

|  | **Control (N=97)** | |  | **PE (N=465)** |  | |
| --- | --- | --- | --- | --- | --- | --- |
|  | **Included (N=35)** | **Excluded (N=62)** | **P value** | **Included (N=191)** | **Excluded (274)** | **P value** |
| Age (years) | 32.7 +/- 5.1 | 32.1 +/- 6.0 | 0.622 | 32.6 +/- 5.5 | 33.6 +/- 5.9 | 0.056 |
| Ethnicity  - Caucasian  - Afro-american  - Hispanic  - Asian | 35 (100%)  0 (0%)  0 (0%)  0 (0%) | 54 (87.1%)  7 (11.2%)  0 (0%)  1 (1.6%) | 0.085 | 118 (61.7%)  38 (19.9%)  31 (16.2%)  4 (2.0%) | 159 (58.6%)  52 (19.1%)  41 (15.1%)  19 (7.0%) | 0.125 |
| BMI (kg/m^2^) | 24.4 +/- 4.3 | 24.0 +/- 3.0 | 0.551 | 29.0 +/- 5.9 | 30.2 +/- 5.1 | **0.013** |
| Smoker | 1 (3.3%) | 0 (0%) | 0.853 | 32 (17.1) | 52 (19.7%) | 0.475 |
| Essential HTN | 0 (0%) | 0 (0%) | NA | 14 (7.4%) | 22 (8.2%) | 0.737 |
| Gestational DM | 0 (0%) | 0 (0%) | NA | 14 (7.4%) | 49 (18.0%) | **0.001** |
| Primiparity | 18 (60.0%) | 24 (48.0%) | 0.298 | 120 (64.5%) | 162 (59.5%) | 0.284 |
| Gestational weeks | 40 (38 – 40) | 39 (38 – 40) | 0.673 | 37 (34 – 39) | 37 (34 – 38) | 0.397 |
| Prior PE | 0 (0%) | 0 (0%) | NA | 14 (7.4%) | 35 (13.5%) | **0.043** |

Bold values indicate p <0.05.

*Abbreviations: PE, preeclampsia; BMI, body mass index; HTN, hypertension; DM, diabetes mellitus.*
